# Case Report: Rhino-orbital Mucormycosis Related to COVID-19: A Case Series Exploring Risk Factors

**DOI:** 10.4269/ajtmh.21-0777

**Published:** 2021-12-13

**Authors:** Sushil Kumar Aggarwal, Upinder Kaur, Dolly Talda, Akshat Pandey, Sumit Jaiswal, Ahalya Kanakan, Anshuman Singh, Sankha Shubhra Chakrabarti

**Affiliations:** ^1^ Department of Otorhinolaryngology, Institute of Medical Sciences, Banaras Hindu University, Varanasi, UP, India;; ^2^ Department of Pharmacology, Institute of Medical Sciences, Banaras Hindu University, Varanasi, UP, India;; ^3^ Department of Obstetrics and Gynaecology, Institute of Medical Sciences, Banaras Hindu University, Varanasi, UP, India;; ^4^ Department of Geriatric Medicine, Institute of Medical Sciences, Banaras Hindu University, Varanasi, UP, India

## Abstract

There has been a surge of rhino-orbital mucormycosis cases in India in the wake of the second wave of the COVID-19 pandemic. It has been widely suggested that dysglycemia resulting from diabetes which is a common comorbidity in COVID-19 patients, and indiscriminate steroid use has resulted in this surge. We report a series of 13 cases of rhino-orbital mucormycosis in COVID-19 patients admitted to our center between mid-April and early June 2021. The cases showed a male preponderance, two patients had loss of vision, and four of them showed intracranial extension of disease. Twelve patients had received steroids and 12 had preexisting or newly diagnosed diabetes, both steroid use and diabetes being the most common identified risk factors. Considering other possible risk factors, immunosuppressed state, antiviral or ayurvedic (Indian traditional) medications, and oxygen therapy were not associated with a definite risk of mucormycosis, because they were not present uniformly in the patients. We propose that COVID-19 itself, through molecular mechanisms, predisposes to mucormycosis, with other factors such as dysglycemia or steroid use increasing the risk.

## INTRODUCTION

The second wave of COVID-19 in India saw an unprecedented surge of mucormycosis cases in its wake, with more than 40,000 cases occurring country-wide.^1^ Mucormycosis is a highly invasive infection caused by fungi of the order Mucorales (e.g., *Rhizopus* sp., *Apophysomyces* sp., *Lichtheimia* sp., *Mucor* sp.), involving most commonly the paranasal sinuses and orbit, with probable intracranial extension. The mortality of mucormycosis is more than 50%, even with treatment, and morbidity in the form of loss of vision is also extremely high.^2^^,^^3^ The exact cause of the increase in mucormycosis cases following COVID-19 is unclear. It has been suggested that COVID-19 patients with diabetes, which is already the leading risk factor for mucormycosis, may have been administered irrational doses of steroids for prolonged periods as a part of COVID-19 management algorithm, and the ensuing dysglycemia may have triggered the mucormycosis epidemic.^4^^,^^5^ We present a series of cases of rhino-orbital mucormycosis and infer the possible risk factors for this fungal epidemic.

## CASE SERIES

We report the case details of 13 patients with rhino-orbital mucormycosis admitted between mid-April and early June 2021 who provided written informed consent (or primary caregivers provided consent if patient incapable) for reporting their cases. The mean age of our patients (10 men, 3 women) was 51.5 years (SD, 10.3 years). Except for one patient, all had a history of diabetes mellitus or were newly diagnosed to have diabetes at the time of presentation for mucormycosis, but none of the patients had evidence of diabetic ketoacidosis. Each patient, barring one, had tested positive for severe acute respiratory syndrome coronavirus 2 on reverse transcriptase–polymerase chain reaction (RT-PCR) performed on a nasal/oropharyngeal swab sample. The one patient who was RT-PCR negative had high-resolution computed tomographic scanning features of the lungs that were highly suggestive for COVID-19. Four patients were detected to be RT-PCR positive after developing symptoms of mucormycosis, and date of test positivity did not imply onset of COVID-19 symptoms, but only timing of presentation at our center. It may be presumed that these patients either had mild or asymptomatic COVID-19, or did not get tested voluntarily for COVID-19 until they presented with bothersome mucormycosis symptoms. One of these patients had mucormycosis symptoms for almost a month before testing RT-PCR positive for severe acute respiratory syndrome coronavirus 2. Prolonged viral shedding may be a possibility in this patient in line with new evidence in immunocompromised patients. Invasive mucormycosis is usually an acute fulminant condition. However, chronic invasive fungal sinusitis may also be caused by Mucorales, even in immunocompetent patients.^6^ Another possibility in this patient is that there was preexisting sinusitis with secondary infection by Mucorales. Twelve of the 13 patients had received steroids, and none of them had developed symptoms of mucormycosis before steroid administration. Rationality of steroid treatment in each patient could not be ascertained. In several cases, steroids had been initiated before presentation to our hospital by trained or untrained local practitioners, and documentation of indication was deficient in such cases. The four patients who received steroids at our center were prescribed the same as part of our COVID-19 management protocol (Table 1). Four patients had intracranial manifestations of disease and two had loss of vision. The demographic and clinical profiles of the patients are detailed in Table 1.

**Table 1:** Profile of patients with rhino-orbital mucormycosis

Age, y/gender	Glycemic status	Other chronic illness	Previous medications*	RT-PCR positivity for SARS-CoV-2	CT severity score (COVID-19)	Onset of symptoms of mucormycosis with respect to RT-PCR positivity	Symptoms pertaining to mucormycosis	Location of mucormycosis (CT scan findings)	Vision	Intracranial involvement	Hematologic parameters	Renal and liver function abnormalities, if any	Fungal diagnosis confirmation	Management^†^	Steroid administered and dose/duration^‡^	Potential risk factors (previously administered medication, etc.)	Outcome; follow-up duration
46/male	H/O diabetes; HbA1c, 9.6	Hypothyroidism	Glimepiride, metformin, thyroxine	Yes	7	30 d before	Nasal blockage, yellowish discharge, right cheek swelling, headache, right peri-orbital pain	Right maxillary and right ethmoid, sphenoid, and frontal sinusitis	Unaffected	No	Hb, 9.5; TLC, 13,300 (N66L27); PLT, 350,000	–	Broad aseptate hyphae on postoperative tissue smear	Right endoscopic debridement of sinuses	Methylprednisolone, 16 mg twice daily for 5 d (before presenting to our center)	Co-amoxiclav, ivermectin, zinc, vitamin C	Discharged alive; 116 d
33/male	Newly diagnosed DM; HbA1c, 8.2	Dyslipidemia	Metformin, teneligliptin, dapagliflozin, fenofibrate, rosuvastatin	No	15	–	Headache, right-side facial swelling	Right maxillary and sphenoid sinusitis	Unaffected	No	Hb, 13.5; TLC, 10,200 (N75L20); PLT, 228,000	–	Broad aseptate hyphae on postoperative tissue smear; CT report was suggestive of fungal sinusitis	Right endoscopic debridement of sinuses	Dexamethasone, 6 mg twice daily for 14 d; hydrocortisone, 100 mg o.d. for 2 d; methylprednisolone, 8 mg o.d. for 7 d (before presenting to our center)	Remdesivir, meropenem, teicoplanin, faropenem, doxycycline, azithromycin, favipiravir, ivermectin, zinc, pirfenidone, vitamin C, oxygen	Discharged alive; 30 d
40/female	Newly diagnosed DM; HbA1c, 9.2	None	Metformin, glimepiride, teneligliptin	Yes	–	9 d before	Pain in and swelling of right eye	Bilateral frontal ethmoid, sphenoid (right > left) sinusitis; right-side optic neuritis	Loss in right eye	Yes	Hb, 10.5; TLC, 11, 230 (N68L25); PLT, 330,000	–	Broad aseptate hyphae on postoperative tissue smear	Bilateral open debridement of sinuses along with orbital exenteration	None	Azithromycin, ivermectin, zinc	Discharged alive; 119 d
47/male	Newly diagnosed DM; HbA1c, 6.8	Hypertension	Insulin, 30/70 pre-mix; amlodipine	Yes	–	16 d after	Right-side facial pain and swelling	Right maxillary and right ethmoid, sphenoid, and frontal sinusitis	Unaffected	No	Hb, 11.9; TLC, 6390 (N68L15); PLT, 210,000	Creatinine, 2; urea, 49.5	Broad aseptate hyphae on preoperative smear; *Mucor *sp. on preoperative culture; granulomatous inflammation on HPE	Right endoscopic debridement of sinuses	Methylprednisolone, 8 mg thrice daily, tapered in 8 d (administered as part of COVID-19 management protocol for cough/dyspnea)	Cefixime, ofloxacin, azithromycin, clarithromycin, ivermectin, zinc, vitamin C	Discharged alive; 78 d
65/male	No dysglycemia; HbA1c, NA	Hypothyroidism	None	Yes	16	18 d before	Swelling of both eyes, headache	Bilateral frontal, maxillary, ethmoid, and sphenoid sinusitis	Unaffected	No	Hb, 9.6; TLC, 6800 (N58L36); PLT, 141,000	-	Broad, aseptate hyphae on postoperative tissue smear	Bilateral open debridement of sinuses	Methylprednisolone, 40 mg thrice daily for 5 d; tapered in 20 d (before presenting to our center)	Azithromycin, moxifloxacin, levofloxacin, meropenem, doxycycline, vitamin C, ivermectin, zinc, fluconazole, oxygen	Discharged alive; 74 d
53/female	H/O diabetes; HbA1c, 8.7	Hypertension	Metformin, telmisartan, warfarin	Yes	–	4 d after	Facial pain and swelling	Right maxillary, ethmoid, and sphenoid sinusitis	Unaffected	No	Hb, 8; TLC, 3200 (N55L37); PLT, 207,000	–	Broad, aseptate hyphae on preoperative smear; *Rhizopus *sp. on preoperative culture; HPE suggestive of mucormycosis	Right-side open debridement of sinuses along with inferior maxillectomy	Dexamethasone, 6 mg twice daily for 12 d (administered as part of COVID-19 management protocol)	Ceftriaxone, cefpodoxime, favipiravir, vitamin C, oxygen	Discharged alive; 112 d
62/male	Newly diagnosed DM; HbA1c, 8.5	Hypertension	Telmisartan, amlodipine	Yes	–	10 d after	Left-side facial swelling, purulent discharge left eye	Left maxillary, ethmoid, frontal sinusitis; mild erosion of left lamina papyracea	Unaffected	No	Hb, 13; TLC, 14,600 (N78L17); PLT, 174,000	–	Broad, aseptate hyphae on preoperative smear; aseptate hyphae on postoperative tissue smear; HPE suggestive of mucormycosis	Left-side open debridement of sinuses	Dexamethasone, 6 mg o.d. for 12 d; methylprednisolone, 125 mg o.d. for 2 d (before presenting to our center)	Methylprednisolone, meropenem, ivermectin, zinc, itraconazole, vitamin C, oxygen	Discharged alive; 64 d
58/male	Newly diagnosed DM; HbA1c, 14.7	None	None	Yes	–	2 d before	Headache, vomiting, right eye pain, delirium	Right maxillary, sphenoid, bilateral ethmoid sinusitis; acute infarct right frontoparietal lobe	Unaffected	Yes	Hb, 14.4; TLC, 8670 (N71L18); PLT, 243,000	–	Broad, aseptate hyphae on postoperative tissue smear	Right-side open debridement of sinuses	Dexamethasone, 4 mg o.d. for 5 d (before presenting to our center)	Doxycycline, azithromycin, ivermectin, ashwagandha (ayurvedic)	Discharged alive; 70 d
40/male	Newly diagnosed DM; HbA1c, 13.5	None	None	Yes	13	18 d after	Headache, facial swelling, pain	Bilateral ethmoid, sphenoid, maxillary sinusitis; extensive orbital and intracranial involvement	Loss in right eye	Yes	Hb, 13; TLC, 14,700 (N86L7); PLT, 300,000	-	Broad, aseptate hyphae on postoperative tissue smear; CT scans suggestive of sinusitis with extensive intracranial involvement	Bilateral open debridement of sinuses, orbital exenteration, craniotomy along with decompression of posterior fossa	Dexamethasone, 8 mg o.d. for 10 d (before presenting to our center)	Gentamicin, meropenem, linezolid	Discharged alive; 110 d
60/male	H/O diabetes; HbA1c, 8.5	Hypertension	Metformin, glimepiride, telmisartan	Yes	–	5 d after	Right facial pain and swelling	Right frontal, ethmoidal sinusitis; soft tissue edema in right periorbital region	Unaffected	No	Hb, 11.2; TLC, 10,600 (N70L20); PLT, 226,000	Creatinine, 1.4; urea, 83	Broad, aseptate hyphae on postoperative tissue smear	Right-side open debridement of sinuses along with orbital decompression	Prednisolone, 30 mg o.d. for 5 d; tapered in 25 d (administered as part of COVID-19 management protocol)	Doxycycline, azithromycin, cefuroxime, high-dose vitamin D, ivermectin, zinc, vitamin C, oxygen	Died after 76 d
65/male	Newly diagnosed DM; HbA1c, 7	Hypertension, coronary artery disease	Cilnidipine, clopidogrel, rosuvastatin, isosorbide dinitrate, tamsulosin, dutasteride	Yes	8	5 d after	Headache, facial pain, eye discharge	Bilateral maxillary, ethmoid, and sphenoid sinusitis; right orbital cellulitis	Unaffected	No	Hb, 10.8; TLC, 5140 (N90L10); PLT, 185,000	-	Fragmented hyphae on postoperative tissue smear; CT indicates sinus involvement with orbital cellulitis	Bilateral open debridement of sinuses, orbital exenteration	Dexamethasone, 2 mg thrice daily for 5 d (before presenting to our center)	Co-amoxiclav, cefixime, ivermectin, zinc, vitamin C; 1 dose of COVAXIN (inactivated SARS-CoV-2 vaccine)	Discharged alive; 120 d
42/male	Newly diagnosed DM; HbA1c, 10.8	None	Insulin	Yes	19	12 d after	Facial swelling and pain, right eye swelling	Right maxillary, sphenoid, and ethmoid sinusitis; right eye involvement	Unaffected	No	Hb, 9.2; TLC, 7060 (N69L20); PLT, 190,000	Creatinine, 2; urea, 45	Broad, aseptate hyphae on postoperative tissue smear; CT scans suggestive	Right open debridement of sinuses, orbital decompression	Prednisolone, 40 mg o.d. for 5 d; methylprednisolone, 8 mg thrice daily for 5 d followed by 16 mg o.d. for 5 d (before presenting to our center)	Piperacillin-tazobactam, cefuroxime, zinc, vitamin C	Discharged alive; 116 d
58/female	H/O diabetes; HbA1c, 12.2	None	None	Yes	14	4 d after	Left facial swelling, left eye swelling	Left maxillary, ethmoid sinusitis; left orbital cellulitis; possible cavernous sinus thrombosis	Unaffected	Yes	Hb, 7.7; TLC, 16,030 (N80L10); PLT, 278,000	–	Broad, aseptate hyphae on postoperative tissue smear	Left open debridement of sinuses, orbital exenteration	Dexamethasone, 8 mg o.d. for 5 d (administered as part of COVID-19 management protocol)	Doxycycline, azithromycin, ivermectin, zinc, vitamin C, oxygen, both doses of COVISHIELD (recombinant adenoviral vaccine for COVID) with the second dose 1 d before PCR positivity	Died after 64 d

COVID-19 = coronavirus disease 2019; CT = computed tomography; DM = diabetes mellitus; Hb = hemoglobin; HbA1c = hemoglobin A1c; H/O = history of; HPE = histopathological examination; NA = not available; o.d. = once daily; PLT = platelets; RT-PCR = reverse transcriptase polymerase chain reaction; SARS-CoV-2 = severe acute respiratory syndrome coronavirus 2; TLC = total leukocyte count. HbA1c is expressed in %; Hb in g/dL; TLC and PLT in /µL

* All patients with dysglycemia were administered insulin while hospitalized. Previous medications refer to hypoglycemic agents and other medications prescribed to them before admission.

^†^ All patients were administered standard antifungal regimens for mucormycosis (amphotericin B/posaconazole) per national guidelines. Remaining surgical management is detailed here.

^‡^ For patients receiving steroids, indication could only be ascertained for those receiving it at our center. Three of four such patients received steroids as part of the COVID-19 management protocol of the institute for moderate to severe cases (hypoxia), along with oxygen therapy; one received steroids for dyspnea and persistent cough (moderate severity). For the remaining eight patients who received steroids before presenting to our center, exact indications could not be ascertained as a result of poor documentation by the local first-contact doctors of these patients (trained/ untrained).  Patient outcomes are indicated until discharge or death.

Potassium hydroxide wet mount and fungal culture/sensitivity were done from nasal swab preoperatively and from tissue samples obtained during operative intervention. Histopathological examination of tissue samples was also performed. Diagnosis of mucormycosis was based on typical clinical presentation supported by positive results in any of these investigations, except in one patient in whom CT scans were suggestive, but a tissue diagnosis/culture report could not be obtained. Each patient received amphotericin B/posaconazole based on national guidelines. The surgical management for each patient and the outcome until death or discharge are detailed in Table 1. There were only two deaths in this series. The low mortality may be because of the involvement of less virulent strains of Mucorales, but this cannot be confirmed in the absence of molecular characterization data.

## DISCUSSION

There have been several reports of mucormycosis cases from India and some from other countries during the COVID-19 crisis, and clinical experience suggests a surge as well. A major review by Dilek et al.^7^ analyzed 30 publications (*N* = 100) with 68 patients from India alone. Corticosteroid use (90.5%) and diabetes (79%) were the major risk factors, and the mortality rate was 33%.^7^ Interestingly, mucormycosis cases were described during the terminal part of the first wave of COVID-19 in India too. One of the earliest series from a major tertiary center in southern India described 10 patients between October and November 2020.^8^ All patients in the series had diabetes, with nine developing ketoacidosis. All 10 patients had also received intravenous dexamethasone as part of the COVID-19 management protocol.^8^

The reasons behind the upsurge of mucormycosis cases in India are still unclear. Experts in mucormycosis research in India hypothesized previously that the high occurrence results from an abundance of Mucorales in the environment as a result of a predominantly hot and humid climate, and a high prevalence of diabetes in Indians.^9^ In the wake of the second wave, mucormycosis cases were reported from most parts of the country. Supplemental Figure 1 shows the area from which our cases presented, but ours being a referral center, it is expected that the usual service region of our hospital would be contributing to the cases. Neglected and undiagnosed diabetes, rather than the absolute duration of diabetes, have been proposed to be risk factors.^9^ It is evident from Table 1 that most patients in our series had been on treatment for diabetes or were newly diagnosed to have diabetes, but they were a mix of patients with uncontrolled and well-controlled blood glucose levels not matching the classically described patients with diabetic ketoacidosis who present with mucormycosis.

Likewise, steroids have been suggested to be risk aggravators, by increasing dysglycemia as well as through their immunosuppressing effect. Even short courses of steroid use have been linked to the occurrence of mucormycosis in susceptible patients.^10^^,^^11^ Other potential risk factors that have been considered include the use of immunomodulators such as tocilizumab, antivirals, ayurvedic (traditional Indian) medicines (especially oils to be instilled in the nose), iron overuse, and use of industrial oxygen.^12^ Increased free iron has been shown to promote the growth of Mucorales in vitro, in mice models, and in patients. The elevated free iron in diabetic ketoacidosis patients impairs interferon-γ production and phagocytic function required for fungal killing.^12^^,^^13^ In line with this, treatment with the iron chelator deferasirox has shown some benefits in clinical outcomes in diabetic patients with mucormycosis.^14^ None of the patients in our series was receiving iron supplements. Host immunosuppression, too, may be thought to predispose individuals to mucormycosis. In the literature, HIV as a risk factor has been seen in 2% of all cases of mucormycosis and in 41% of those who succumb to the fungal illness.^15^ In HIV patients with mucormycosis, intravenous drug use, neutropenia, and corticosteroid use are the common precipitating factors.^16^ However, no patient in our series had HIV infection.

A look at Table 1 suggests that none of the tentative factors, including steroids and diabetes, were present uniformly in the COVID-19 patients presenting with rhino-orbital mucormycosis, although diabetes and steroid use were present in the majority. Only 6 of 13 patients had received oxygen at some point, and only one patient had been taking ayurvedic oral medicines (containing ashwagandha, *Withania somnifera*). There may be other unexplored factors, such as patient age and presence of chronic kidney disease, that may determine risk. Literature pertaining to this is varied. Because mucormycosis is usually secondary to diverse immunosuppressing conditions, cases expectedly follow the age trends of these conditions. The sample size in our case series was small, so comments cannot be made on age predilection of the disease. Two of the patients in our series had renal dysfunction with mildly elevated serum creatinine, but because of coexisting diabetes, the role of kidney disease as a risk factor cannot be commented upon.

It is quite likely that any of the studied factors such as dysglycemia or steroid use might have played only a facilitatory role in triggering mucormycosis cases in COVID-19 patients. There may be molecular associations between the two infectious entities that provide the primary predisposition. COVID-19 has been observed to increase serum concentrations of GRP78, a heat-shock protein involved in stress responses.^17^ GRP78 has been demonstrated to bind to *Rhizopus* germlings, which are the major invading forms of Mucorales.^18^ Furthermore, antibodies directed against GRP78 and short hairpin RNA sequences targeting GRP78 have been observed to suppress invasion and endothelial damage induced by *Rhizopus delemar*, but not by other pathogenic fungi such as *Candida* and *Aspergillus*.^18^^,^^19^ Interestingly, GRP78 associates with angiotensin-converting enzyme 2 and S (spike) protein of severe acute respiratory syndrome coronavirus 2, facilitating the viral entry into host cells.^20^ In line with this, anti-GRP78 antibodies have been suggested as potential COVID-19 therapeutic options.^21^ This may have a dual effect and may reduce the risk of mucormycosis as well. A second link between COVID-19 and mucormycosis involves spleen tyrosine kinase, an enzyme involved in phagocytic function of neutrophils, macrophages, and so on, hence playing a major role in antifungal defense. Urine proteome analysis of COVID-19 patients has shown a downregulation of spleen tyrosine kinase, which may impair phagocytosis and predispose to invasive mucormycosis.^22^ Paradoxically, fostamatinib, a small molecule inhibitor of spleen tyrosine kinase, is being tested in clinical trials of COVID-19 patients by virtue of its inhibitory effect on pro-inflammatory cytokines and the neutrophil extracellular trap.^23^ In contrast to therapies targeting GRP78, this drug may increase the risk of invasive mucormycosis by inhibiting the phagocytic antifungal defense of the body. However, fostamatinib is still an investigational therapy in COVID-19 and was not administered to any of our patients.

Our center saw 1,828 COVID-19 admissions between January 1, 2021 and August 21, 2021. During the same period, there were 280 admissions of mucormycosis cases, mostly between May and July. Our center is a referral center for both conditions, and the number of referral centers for mucormycosis was much less compared with that for COVID-19. The number of admissions represent disparate sets of patients, and it may not be possible to draw conclusions regarding the incidence of mucormycosis in COVID-19 patients from this. Approximately 10% of mucormycosis cases at our hospital were those who underwent COVID-19 management here and subsequently developed the fungal infection. Overall, mucormycosis cases are rare even in COVID-19 patients, although a surge has been undeniable in the wake of the second wave in India. It is possible that COVID-19—through molecular mechanisms involving GRP78, SYK, and other cellular immune factors—creates a favorable environment for mucormycosis.^12^ The interplay between the two infections needs to be worked out based on these pathogenetic pathways to prevent invasive fungal disease with high mortality.

## Supplemental Material


Supplemental materials

## References

[1] Raut A Huy NT, 2021. Rising incidence of mucormycosis in patients with COVID-19: another challenge for India amidst the second wave? *Lancet Respir Med. 9:* e77. 10.1016/S2213-2600(21)00265-4 PMC8175046 34090607

[2] Palejwala S Zangeneh T Goldstein S Lemole GM, 2016. An aggressive multidisciplinary approach reduces mortality in rhinocerebral mucormycosis. Surg Neurol Int 7: 61. 27280057 10.4103/2152-7806.182964 PMC4882964

[3] Liang KP Tleyjeh IM Wilson WR Roberts GD Temesgen Z, 2006. Rhino-orbitocerebral mucormycosis caused by *Apophysomyces elegans* . J Clin Microbiol 44: 892–898. 16517873 10.1128/JCM.44.3.892-898.2006 PMC1393113

[4] Hoenigl M , 2021. The emergence of COVID-19 associated mucormycosis: analysis of cases from 18 countries. SSRN Electron J. DOI: 10.2139/ssrn.3844587. PMC8789240 35098179

[5] Tabarsi P Khalili N Pourabdollah M Sharifynia S Safavi Naeini A Ghorbani J Mohamadnia A Abtahian Z Askari E, 2021. Case report: COVID-19-associated rhinosinusitis mucormycosis caused by *Rhizopus arrhizus*: a rare but potentially fatal infection occurring after treatment with corticosteroids. Am J Trop Med Hyg 105: 449–453. 34237015 10.4269/ajtmh.21-0359 PMC8437195

[6] Alotaibi NH Omar OA Altahan M Alsheikh H Al Mana F Mahasin Z Othman E, 2020. Chronic invasive fungal rhinosinusitis in immunocompetent patients: a retrospective chart review. Front Surg 7: 608342. 10.3389/fsurg.2020.608342 PMC7772145 33392248

[7] Dilek A Ozaras R Ozkaya S Sunbul M Sen EI Leblebicioglu H, 2021. COVID-19-associated mucormycosis: case report and systematic review. Travel Med Infect Dis 44: 102148. 34454090 10.1016/j.tmaid.2021.102148 PMC8387131

[8] Sarkar S Gokhale T Choudhury S Deb A, 2021. COVID-19 and orbital mucormycosis. Indian J Ophthalmol 69: 1002–1004. 33727483 10.4103/ijo.IJO_3763_20 PMC8012924

[9] Prakash H Chakrabarti A, 2021. Epidemiology of mucormycosis in India. Microorganisms 9: 523. 33806386 10.3390/microorganisms9030523 PMC8000977

[10] Hoang K Abdo T Reinersman JM Lu R Higuita NIA, 2020. A case of invasive pulmonary mucormycosis resulting from short courses of corticosteroids in a well-controlled diabetic patient. Med Mycol Case Rep 29: 22–24. 32547914 10.1016/j.mmcr.2020.05.008 PMC7286928

[11] Ferguson AD, 2007. Rhinocerebral mucormycosis acquired after a short course of prednisone therapy. J Am Osteopath Assoc 107: 491–493. 18057223

[12] Chakrabarti SS Kaur U Aggarwal SK Kanakan A, 2021. The pathogenetic dilemma of post-COVID-19 mucormycosis in India. *Aging Dis.* DOI: 10.14336/AD.2021.0811. PMC8782544 35111359

[13] Ibrahim AS, 2011. Host cell invasion in mucormycosis: role of iron. Curr Opin Microbiol 14: 406–411. 21807554 10.1016/j.mib.2011.07.004 PMC3159741

[14] Spellberg B Andes D Perez M Anglim A Bonilla H Mathisen GE Walsh TJ Ibrahim AS, 2009. Safety and outcomes of open-label deferasirox iron chelation therapy for mucormycosis. Antimicrob Agents Chemother 53: 3122–3125. 19433555 10.1128/AAC.00361-09 PMC2704638

[15] Roden MM , 2005. Epidemiology and outcome of zygomycosis: a review of 929 reported cases. Clin Infect Dis 41: 634–653. 16080086 10.1086/432579

[16] Moreira J Varon A Galhardo MC Santos F Lyra M Castro R Oliveira R Lamas CC, 2016. The burden of mucormycosis in HIV-infected patients: a systematic review. J Infect 73: 181–188. 27394402 10.1016/j.jinf.2016.06.013

[17] Sabirli R Koseler A Goren T Turkcuer I Kurt O, 2021. High GRP78 levels in COVID-19 infection: a case-control study. Life Sci 265: 118781. 33220289 10.1016/j.lfs.2020.118781 PMC7674149

[18] Liu M Spellberg B Phan QT Fu Y Fu Y Lee AS Edwards JE Filler SG Ibrahim AS, 2010. The endothelial cell receptor GRP78 is required for mucormycosis pathogenesis in diabetic mice. J Clin Invest 120: 1914–1924. 20484814 10.1172/JCI42164 PMC2877958

[19] Baldin C Ibrahim AS, 2017. Molecular mechanisms of mucormycosis: the bitter and the sweet. PLOS Pathog 13: e1006408. 28771587 10.1371/journal.ppat.1006408 PMC5542377

[20] Carlos AJ Ha DP Yeh D-W Van Krieken R Tseng C-C Zhang P Gill P Machida K Lee AS, 2021. The chaperone GRP78 is a host auxiliary factor for SARS-CoV-2 and GRP78 depleting antibody blocks viral entry and infection. J Biol Chem 296: 100759. 33965375 10.1016/j.jbc.2021.100759 PMC8102082

[21] Ha DP Van Krieken R Carlos AJ Lee AS, 2020. The stress-inducible molecular chaperone GRP78 as potential therapeutic target for coronavirus infection. J Infect 81: 452–482. 10.1016/j.jinf.2020.06.017 PMC7289740 32535155

[22] Tian W , 2020. Immune suppression in the early stage of COVID-19 disease. Nat Commun 11: 5859. 33203833 10.1038/s41467-020-19706-9 PMC7673112

[23] Strich JR Ramos-Benitez MJ Randazzo D Stein SR Babyak A Davey RT Suffredini AF Childs RW Chertow DS, 2021. Fostamatinib inhibits neutrophils extracellular traps induced by COVID-19 patient plasma: a potential therapeutic. J Infect Dis 223: 981–984. 33367731 10.1093/infdis/jiaa789 PMC7799006

