# Supplementary figures and images for: Case Report: Rhino-orbital Mucormycosis Related to COVID-19: A Case Series Exploring Risk Factors
Source: Am J Trop Med Hyg. 2021 Dec 13;106(2):566–70. doi: 10.4269/ajtmh.21-0777 (PMC8832906; doi:10.4269/ajtmh.21-0777)

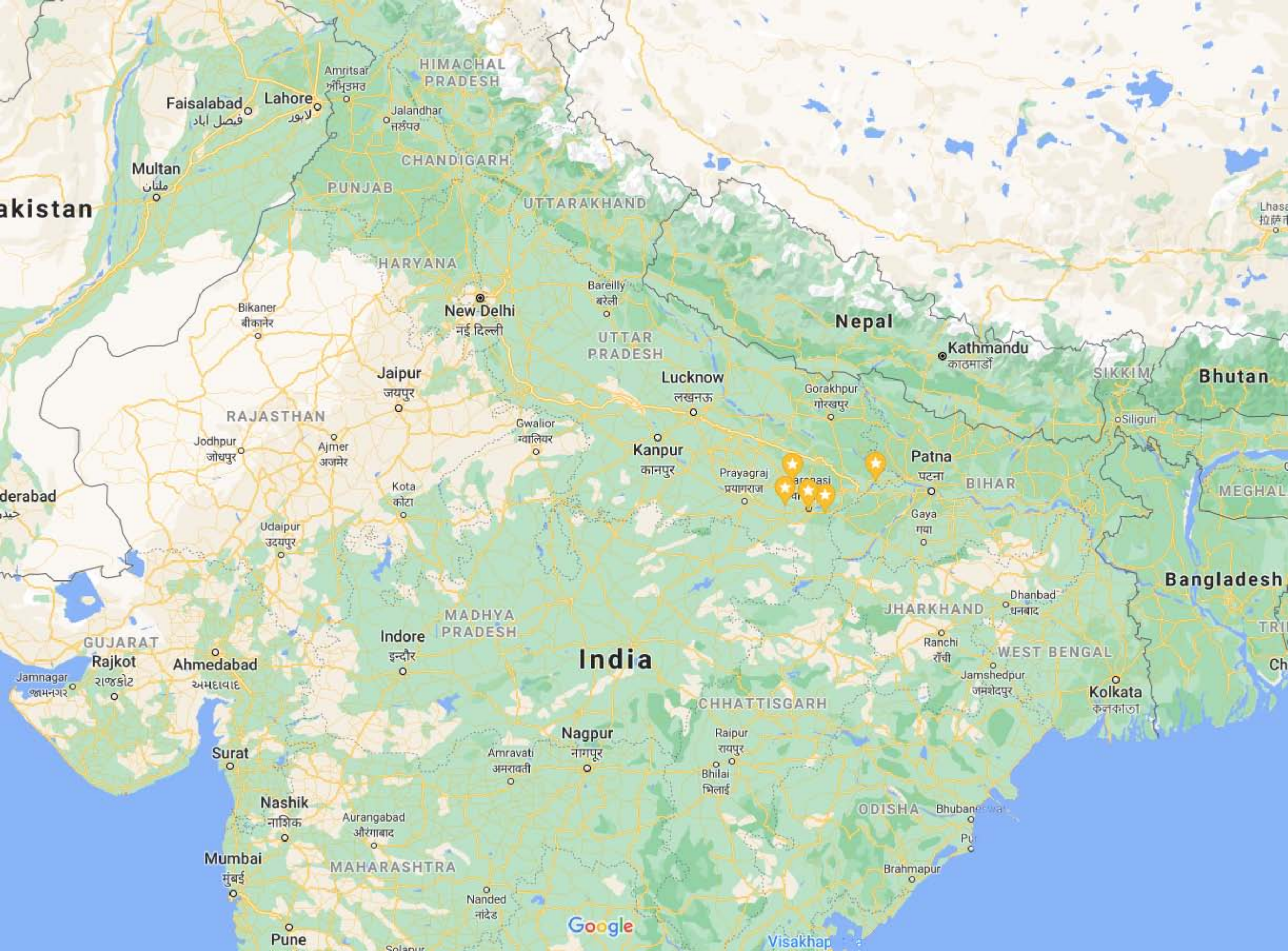

Supplement: Supplementary file 1 [file tpmd210777.SD1.pdf]
